# Predictors of iatrogenic splenic injury in radical gastrectomy for gastric cancer

**DOI:** 10.3389/fonc.2024.1361185

**Published:** 2024-03-27

**Authors:** Xin Zhang, Ziran Wei, Hongbing Fu, Zunqi Hu, Weijun Wang, Ronglin Yan

**Affiliations:** Department of Gastrointestinal Surgery, Second Affiliated Hospital of Naval Medical University, Shanghai, China

**Keywords:** gastric cancer, iatrogenic splenic injury, gastrectomy, surgical complication, retrospective study

## Abstract

**Background:**

Iatrogenic splenic injury (ISI) is a recognized complication in radical gastrectomy that may result in incidental splenectomy (IS). However, the predictors of such events remain largely unknown.

**Methods:**

Medical records of the patients who underwent radical gastrectomy at our institution between January 2015 and December 2022 were retrospectively reviewed. Potential predictors of ISI and IS were collected and analyzed by multivariate logistic regression. Results were reported as an odds ratio (OR) with 95% confidence intervals (CI).

**Results:**

A total of 2916 patients were included, of whom 211 patients (7.2%) suffered from ISI and 75 patients (2.6%) underwent IS. Multivariate analysis demonstrated that BMI≥25 (OR: 3.198 (2.356-4.326), p<0.001), total gastrectomy (OR: 2.201 (1.601-3.025), p<0.001), and the existence of “criminal fold” (OR: 13.899 (2.824-251.597), p=0.011) were independent predictive risk factors for ISI; whereas laparoscopic surgical approach (OR: 0.048 (0.007-0.172), p<0.001) was a protective factor for ISI. Moreover, the existence of “criminal fold” (OR: 15.745 (3.106-288.470), p=0.008) and BMI≥25 (OR: 2.498 (1.002-6.046), p=0.044) were identified as independent risk factors of ISI under laparoscopic gastrectomy. There was no association between sex, age, previous abdominal surgery, neoadjuvant therapy, outlet obstruction, tumor stage, nodal stage, and total lymph node retrieved and ISI.

**Conclusions:**

BMI≥25 and total gastrectomy can predict high risk of ISI during radical gastrectomy. Laparoscopic surgery is superior to open gastrectomy in lowing the risk of ISI.

## Introduction

Gastric cancer (GC) is one of the most common malignancies and a leading cause of cancer mortality worldwide (1). Radical gastrectomy with concomitant lymph node dissection is deemed as the optimal treatment to achieve clinical cure for GC (2). However, this procedure is not exempt from complications. Iatrogenic splenic injury (ISI) is a recognized complication in abdominal surgery that may result in incidental splenectomy (IS), which further increases operation time, hospital stay, patient morbidity and mortality (3). Moreover, iatrogenic splenic lesions with splenectomy were identified as a potential risk factor for a worse oncological long-term outcome for cancer patients (4). However, few studies have been conducted to analyze ISI during radical gastrectomy, and the incidence of ISI and IS during radical gastrectomy is believed to be underestimated due to poor documentation. More importantly, it is still unclear how this type of injury occurred, which makes it difficult to evaluate medical professional liability.

To address this gap in knowledge, we attempt to determine the predisposing factors for ISI and IS during radical gastrectomy for GC based on single-institutional medical records. These results may help the surgical team foresee the patients at high risk for ISI, guide the selection of optimal treatment modality, institute risk-reduction strategies, and properly inform patients of the risks prior to surgery.

## Methods

### Definitions

In this study, ISI is defined as any unintentional rupture of the splenic capsule (peritoneum) or splenic laceration with bleeding. It is classified as laceration, capsular tear, and rupture.

IS is defined as any splenectomy procedure performed in conjunction with stomach resection except a preoperatively intended splenectomy.

“Criminal fold” is defined as a definite peritoneal band running medially from the lower pole of the spleen to the great omentum, adjacent to the greater curvature of the stomach.

### Study population

Medical records of the patients who underwent radical gastrectomy at the Second Affiliated Hospital of Naval Medical University between January 2015 and December 2022 were retrospectively reviewed. The inclusion criteria were as follows: 1) pathologically diagnosed as adenocarcinoma, mucinous adenocarcinoma, signet-ring cell carcinoma, neuroendocrine carcinoma, and so on; 2) radical gastrectomy with D1, D1+, D2 or D2+ lymphadenectomy. Patients fulfilling any of the exclusion criteria were excluded: 1) pathologically diagnosed as gastric benign ulcer or stromal tumor; 2) existence of distant metastasis including retroperitoneal lymph node, left supraclavicular lymph node, liver, lung or bone metastasis; 3) splenectomized before the stomach surgery; 4) splenectomy for primary splenic disease or trauma; 5) gastrectomy in combination of other organs for oncologic reason, such as spleen, partial liver or pancreas, colon, or intestine; 6) gastrectomy for remnant gastric cancer; 7) gastrectomy without lymph node dissection.

This study was approved by the ethics committee of the second affiliated hospital of naval medical university (No. 2023SL006)

### Data collection

The relevant clinical and pathological data of each included patient were extracted by reviewing the medical records, operative notes, pathological examination records, and laparoscopic surgery videos (if available). The potential predictors of ISI or IS cover three aspects, namely patient characteristics, tumor characteristics and surgical information. The specific variables were as follows: sex (female or male), age, body mass index (BMI) ≥25 (yes or no), outlet obstruction (yes or no), neoadjuvant therapy (yes or no), pathological tumor stage (T0, Tis, T1, T2, T3, T4a) and nodal stage (N0, N1, N2, N3a, N3b), previous abdominal surgery (yes or no), surgical approach (open or laparoscopic), type of procedure (distal gastrectomy, proximal gastrectomy, total gastrectomy), total lymph node retrieved (TLNR). The tumor and nodal stage were determined based on the 8^th^ American joint committee on cancer (AJCC) staging system. Specially, the results of the ypTNM stage were used as a replacement for the pathological tumor and nodal stage if the patients had received neoadjuvant therapy. T0 stage was documented when pathological complete remission was achieved.

Laparoscopic surgery videos were reviewed for some details on the procedure when the video documents were available. Specially, whether or not a “criminal fold” attached to the spleen existed were checked and documented (yes or no). Procedures that were started as laparoscopic, but were converted to open before the splenic injury occurred were classified as open; while those that were converted to open after the injury occurrence as laparoscopic.

### Statistical analysis

Continuous variables were presented as mean ± standard deviation for normal distributed data, and as median with interquartile range (IQR) for skewed distributed data. Intergroup differences for continuous variables were compared using the Student’s t-test or non-parametric Mann-Whitney U test, as appropriate. Categorical variables were presented as frequency with percentage. The Chi-square and Fisher’s exact tests (when appropriate) were used for intergroup comparison of nominal categorical variables. Exposure to each of the predefined predictors listed above in relation to the risk of ISI or IS was analyzed using multivariate logistic regression, which provided adjusted odds ratios (ORs) with 95% confidence intervals (CIs). A numerical transformation was performed for the two ordinal categorical variables (tumor stage and nodal stage), and the intergroup comparison and regression analysis were in accordance with that for continuous variables. All statistical analyses were performed by R software (version 4.1.3, https://www.r-project.org/). For all statistical tests, a two-sided p value less than 0.05 was regarded as statistically significant.

## Results

### Baseline characteristics

A total of 2916 patients were finally included in the data analysis, of whom 211 patients (7.2%) suffered from ISI and the remaining 2705 patients had no ISI. Among the 211 patients with ISI, spleen preserving procedures, such as compression, primary repair with electrocautery and/or topical hemostatic agents, or splenorraphy were performed for 136 ISI patients; while IS was ultimately performed for 75 patients due to inadequate hemostasis and ongoing bleeding. The IS rate for the overall radical gastrectomy in our institution is 2.6%. In open surgery subgroup, the average operation time and blood loss is 171.6min and 110.4ml. In laparoscopic surgery subgroup, the average operation time and blood loss is 213.4min and 87.0ml. Detailed comparisons of the potential predictors for ISI and IS are shown in **Table 1**.

**Table 1:** Characteristics in relation to iatrogenic splenic injury and incidental splenectomy in study population.

	Iatrogenic splenic injury			Incidental splenectomy		
	No (n=2705)	Yes (n=211)	p value	No (n=2841)	Yes (n=75)	p value
Sex, n (%)			0.123			0.854
Female	796 (29.4)	51 (24.2)		824 (29.0)	23 (30.7)	
Male	1909 (70.6)	160 (75.8)		2017 (71.0)	52 (69.3)	
Age (median (IQR))	62.0 (54.0-68.0)	64.0 (56.0-70.0)	0.033	62.0 (54.0-68.0)	65.0 (56.0-69.5)	0.077
BMI≥25, n (%)			<0.001			<0.001
No	2218 (82.0)	127 (60.2)		2300 (81.0)	45 (60.0)	
Yes	487 (18.0)	84 (39.8)		541 (19.0)	30 (40.0)	
Neo, n (%)			1			1
No	2455 (90.8)	191 (90.5)		2578 (90.7)	68 (90.7)	
Yes	250 (9.2)	20 (9.5)		263 (9.3)	7 (9.3)	
Tumor stage, n (%)			<0.001			0.002
T0	16 (0.6)	2 (0.9)		17 (0.6)	1 (1.3)	
Tis	71 (2.6)	5 (2.4)		74 (2.6)	2 (2.7)	
T1	671 (24.8)	27 (12.8)		693 (24.4)	5 (6.7)	
T2	219 (8.1)	14 (6.6)		228 (8.0)	5 (6.7)	
T3	507 (18.7)	31 (14.7)		522 (18.4)	16 (21.3)	
T4a	1221 (45.1)	132 (62.6)		1307 (46.0)	46 (61.3)	
Nodal stage, n (%)			<0.001			0.103
N0	1115 (41.5)	65 (30.8)		1154 (40.9)	26 (34.7)	
N1	463 (17.2)	36 (17.1)		487 (17.3)	12 (16.0)	
N2	478 (17.8)	36 (17.1)		502 (17.8)	12 (16.0)	
N3a	442 (16.4)	47 (22.3)		473 (16.8)	16 (21.3)	
N3b	189 (7.0)	27 (12.8)		207 (7.3)	9 (12.0)	
TLNR (median (IQR))	22.0 (17.0-29.0)	24.0 (18.0-31.0)	0.062	22.0 (17.0-29.0)	22.0 (16.0-31.5)	0.929
Type of procedure, n (%)			<0.001			<0.001
Distal	1819 (67.2)	101 (47.9)		1898 (66.8)	22 (29.3)	
Proximal	193 (7.1)	20 (9.5)		203 (7.1)	10 (13.3)	
Total	693 (25.6)	90 (42.7)		740 (26.0)	43 (57.3)	
Surgical approach, n (%)			<0.001			0.032
Open	2015 (74.5)	187 (88.6)		2137 (75.2)	65 (86.7)	
Laparoscopic	690 (25.5)	24 (11.4)		704 (24.8)	10 (13.3)	
Outlet obstruction, n (%)			0.081			1
No	2656 (98.2)	203 (96.2)		2785 (98.0)	74 (98.7)	
Yes	49 (1.8)	8 (3.8)		56 (2.0)	1 (1.3)	
Previous abdominal surgery, n (%)			0.201			1
No	2474 (91.5)	187 (88.6)		2593 (91.3)	68 (90.7)	
Yes	231 (8.5)	24 (11.4)		248 (8.7)	7 (9.3)	
Criminal fold, n (%)			0.005			0.178
No	142 (5.2)	1 (0.5)		142 (5.0)	1 (1.3)	
Yes	214 (7.9)	22 (10.4)		227 (8.0)	9 (12.0)	
Missing	2349 (86.8)	188 (89.1)		2472 (87.0)	65 (86.7)	

IQR, interquartile range; BMI, body mass index; TLNR, total lymph node retrieved.

### Predictors of ISI and IS during radical gastrectomy

Multivariate logistic analysis showed that sex, age, previous abdominal surgery, neoadjuvant therapy, outlet obstruction, tumor stage, nodal stage, and TLNR had no significant influence on the risk of either ISI or IS, although neoadjuvant therapy showed an obvious tendency to increase the risk of ISI (OR: 1.672, 95% CI: 0.973-2.746). BMI≥25 significantly increased the risk of both ISI and IS (OR: 3.198, 95% CI: 2.356-4.326; OR: 3.118, 95% CI: 1.903-5.045, respectively), whereas laparoscopic surgery significantly reduced the risk of both ISI and IS in comparison with open surgery (OR: 0.048, 95%CI: 0.007-0.172; OR: 0.274, 95% CI: 0.001-0.272, respectively). For the type of procedure, total gastrectomy was associated with increased risk of both ISI and IS (OR: 2.201, 95% CI: 1.601-3.025; OR: 4.845, 95% CI: 2.840-8.500, respectively), while proximal gastrectomy significantly increased the risk of IS (OR: 3.321, 95% CI: 1.452-7.126) and marginally increase the risk of ISI (OR: 1.610, 95% CI: 0.929-2.675). The existence of criminal fold independently predicts the incidence of ISI (OR: 13.899, 95% CI: 2.824-251.597), but had no obvious influence on the risk of IS. The logistic regression results were summarized in **Table 2**.

**Table 2:** Predictors of ISI and IS during radical gastrectomy.

	ISI (n=211)			IS (n=75)		
	OR	95% CI	p value	OR	95% CI	p value
Sex						
Male	1.000	Reference		1.000	Reference	
Female	0.844	0.595-1.178	0.328	1.315	0.768-2.189	0.304
Age	1.011	0.997-1.026	0.112	1.015	0.993-1.038	0.193
BMI≥25						
No	1.000	Reference		1.000	Reference	
Yes	3.198	2.356-4.326	<0.001	3.118	1.903-5.045	<0.001
Previous abdominal surgery						
No	1.000	Reference		1.000	Reference	
Yes	1.285	0.791-2.008	0.290	0.979	0.399-2.055	0.959
Neoadjuvant therapy						
No	1.000	Reference		1.000	Reference	
Yes	1.672	0.973-2.746	0.051	1.548	0.617-3.354	0.305
Outlet obstruction						
No	1.000	Reference		1.000	Reference	
Yes	2.042	0.852-4.354	0.083	0.862	0.048-4.264	0.886
Tumor stage	1.145	0.996-1.323	0.061	1.232	0.977-1.581	0.088
Nodal stage	1.078	0.957-1.215	0.215	0.990	0.817-1.201	0.922
Type of procedure						
Distal gastrectomy	1.000	Reference		1.000	Reference	
Proximal gastrectomy	1.610	0.929-2.675	0.076	3.321	1.452-7.126	0.003
Total gastrectomy	2.201	1.601-3.025	<0.001	4.845	2.840-8.500	<0.001
Surgical approach						
Open	1.000	Reference		1.000	Reference	
Laparoscopic	0.048	0.007-0.172	<0.001	0.274	0.001-0.272	0.018
TLNR	0.999	0.984-1.014	0.945	0.985	0.960-1.010	0.262
Criminal fold						
No	1.000	Reference		1.000	Reference	
Yes	13.899	2.824-251.597	0.011	4.846	0.872-9.072	0.140
Missing	0.559	0.048-12.596	0.647	0.095	0.002-3.722	0.196

OR, odds ratio; CI, confidence interval; BMI, body mass index; TLNR, total lymph node retrieved.

### Predictors of ISI during laparoscopic radical gastrectomy

Laparoscopic gastrectomy was performed in 714 of all the 2916 patients, while open gastrectomy was performed in the remaining 2202 patients. Among the 714 laparoscopic gastrectomies, ISI and IS occurred in 24 (3.4%) and 10 (1.4%) procedures, respectively. Video documents were available for 378 cases to provide sufficient information to evaluate the existence of the “criminal fold”. Multivariate logistic analysis performed on the 714 laparoscopic radical gastrectomies demonstrated that only the existence of “criminal fold” and BMI≥25 were identified as independent risk factors of ISI under laparoscopic setting (**Table 3**). Since only 10 IS cases in the study cohort, we did not perform the multivariate logistic analysis for IS in laparoscopic radical gastrectomy.

**Table 3:** Predictors of ISI during laparoscopic radical gastrectomy.

	Iatrogenic splenic injury (n=24)		
	OR	95% CI	p value
Sex			
Male	1.000	Reference	
Female	1.495	0.560-3.745	0.401
Age	0.962	0.918-1.009	0.105
BMI≥25			
No	1.000	Reference	
Yes	2.498	1.002-6.046	0.044
Previous abdominal surgery			
No	1.000	Reference	
Yes	2.025	0.523-6.477	0.261
Neoadjuvant therapy			
No	1.000	Reference	
Yes	0.309	0.016-1.690	0.272
Outlet obstruction			
No	1.000	Reference	
Yes	3.048	0.131-32.973	0.386
Tumor stage	1.346	0.860-2.134	0.198
Nodal stage	0.810	0.503-1.229	0.348
Type of procedure			
Distal gastrectomy	1.000	Reference	
Proximal gastrectomy	1.400	0.197-6.194	0.689
Total gastrectomy	1.079	0.335-3.270	0.894
TLNR	0.981	0.922-1.038	0.522
Criminal fold			
No	1.000	Reference	
Yes	15.745	3.106-288.470	0.008
Missing	0.463	0.018-11.912	0.590

ISI, iatrogenic splenic injury; OR, odds ratio; CI, confidence interval;

BMI, body mass index; TLNR, total lymph node retrieved.

## Discussion

ISI is defined as any unintentional damage caused to the spleen by the surgeon or the assistant(s) during a surgical procedure (5). It can occur during abdominal surgery due to damage caused by thermic injury, excessive traction, and/or misplaced retractors (6). The true incidence of iatrogenic splenic injuries is difficult to assess due to variability in reporting and documentation (7). Nevertheless, there has been no specialized study exploring the predictors of the iatrogenic splenic injury during radical gastrectomy for gastric cancer. We found that patient BMI≥25, total gastrectomy procedure, surgical approach applied, and the existence of “criminal fold” are independent predictors of ISI. Laparoscopic surgery is superior to open gastrectomy in lowing the risk of ISI and IS. Knowledge of these risk factors will help surgeons in their decision-making process and in properly informing patients regarding their risks.

Currently, laparoscopy is recommended as a routine surgical technique for the resection of gastrointestinal cancer since large-scale prospective studies have shown that laparoscopic surgery was equivalent to open surgery in terms of safety and long-term prognosis (8–10). A number of studies have shown that laparoscopic colon resection is superior to open surgery on the incidence of ISI and IS (11, 12). In accordance with the literature, we found that gastrectomy performed laparoscopically was less associated with ISI and IS compared with laparotomic procedure. Possible advantages of laparoscopy include superior visualization and better exposure of the spleen and its attachments, which help the surgeons avoid using unnecessary traction during procedures (13). Additionally, with the popularity of laparoscopic gastrectomy, the use of retractors has become less and less, which may further translate into less ISI caused by retractors in open procedure.

Radical gastrectomy has currently been accepted as the mainstay in the treatment of GC (14). During the procedure, the spleen may be injured in three ways: excessive traction, application of retractors or directly by the surgical instruments (15). Therefore, splenic injury can be reduced by avoiding undue traction, achieving good exposure, and careful division of splenic ligaments and adhesions (16). Especially, thorough familiarity with the anatomy of the spleen and its attachments, attention to established principles of technique and exposure, and recognition of those patients especially susceptible to splenic injury will help reduce the incidence of this event (17). Derogar et al (18) found an inverse association between surgeon volume and ISI in esophageal cancer surgery. Moreover, teaching hospital was associated with a higher risk of splenic injury due to the inexperience of the trainees involved with the operations (19). These results indicate that surgical experience plays a critical role in the prevention of ISI. Similarly, the rate of ISI and IS also showed a tendency to decrease in more recent cases in our study (data not shown). This may be attributed to both the accumulating surgical experience of the surgeon and the popularity of the laparoscopic surgery.

The extent and dissection scope of an operation may have an influence on the incidence of ISI. The analysis from our series confirmed that the extent of the resection of the stomach was a predictor of ISI. Total gastrectomy had a higher risk of ISI than distal gastrectomy, which is not unexpected in view that a greater extent of perisplenic division is needed in total gastrectomy than that in partial gastrectomy. Another, the lymph node dissection is believed to be a technically difficult and risky procedure. However, TLNR did not show a relationship with the risk of ISI in our series, indicating that the extent of the lymph node dissection seemed not to be a predictor of ISI. These results were supported by the recent literatures showing the safety and feasibility of laparoscopic lymph node dissection in the procedure of radical gastrectomy (20, 21).

Since previous abdominal surgery may develop dense adhesions in the left upper quadrant of the abdomen, traction on various structures indirectly may result in traction on the splenic capsule through these adhesions, which might be an important cause of ISI (6). Another, difficult dissection of these adhesions to obtain exposure and to free structures may also bring about direct injury to the spleen. Therefore, reoperation is believed to be an important factor predisposing the spleen to iatrogenic injury in the era of open procedure (5). In contrast, our present study showed that the risk of ISI during radical gastrectomy procedure was not significantly increased in patients who had previously undergone abdominal surgery. This might be explained by the fact that a majority of the previous surgeries, such as appendicectomies or cholecystectomies, did not involve dissection in the left upper quadrant, which thus would not cause severe perisplenic adhesions. Moreover, the application of laparoscopy would help provide a better exposure of the whole peritoneal cavity, and perisplenic adhesions could be dissected before undue traction tearing the splenic capsule.

It is interesting to note that the existence of “criminal fold” is an independent risk factor of ISI during laparoscopic radical gastrectomy. When it comes to the “criminal fold”, Lord et al. firstly demonstrated such a peritoneal band in a large series of autopsy specimens, which accounts for the susceptibility of the lower pole of the spleen to avulsion injury in gastric and colonic surgery (22). The definition of “criminal fold” was first proposed by Millikan (23). More recently, Wang et al (24) revealed that “criminal fold” existed in 81.5% cases, and ISI mostly resulted from improper traction of “criminal fold” during laparoscopic gastrectomy. Therefore, they suggested that division of the “criminal fold” should be a priority before the division of the left half of the gastrocolic omentum. In the present study, we found that “criminal fold” existed in 62.2% (235/378) cases, and patients with “criminal fold” have an estimated over thirteen times higher risk for ISI. Since data of the existence of “criminal fold” were missing in 86.8% of the included patients, it is not reliable enough to come to a firm conclusion. Even so, these results indicate that attention should be paid to the existence of “criminal fold” during the division of the greater curvature of the stomach, and the priority division of the “criminal fold” is highly recommended to reduce the risk of ISI.

Patient and disease characteristics may have a bearing on the risk of ISI. Obesity was believed to increase the risk of perioperative complications, since too much visceral fat would bring about difficulty in achieving adequate exposure (25). Accordingly, BMI and visceral fat area (VFA) were proposed as predictive factors for intraoperative complication during laparoscopic gastrectomy in previous literatures (26–28). Similarly, we also found that BMI≥25 was associated with higher risk of both ISI and IS. Another, advancing age has been believed to increase the friability of the spleen secondary to degenerative vascular disease, as well as lack of rib elasticity resulting in over vigorous retraction of the left costal margin (5). However, we failed to show any association between age and the incidence of ISI and IS in the present study. Moreover, patient sex, tumor stage, nodal stage, neoadjuvant therapy or outlet obstruction did not have an independent impact on the incidence of ISI and IS.

Some limitations need to be clarified in this study. 1) Although laparoscopic radical gastrectomy has been recommended in most of the cases, open surgeries are still needed in patients with previous abdominal surgery, severe or advanced primary disease. Therefore, factors affecting the surgeons’ decision about the surgical approach may bias this finding. 2) Inaccurate documentation of the indication for IS cannot be completely ruled out. Especially, it is likely that some surgeons might provide “oncological explanations” for what would be otherwise a splenectomy not justified by the primitive disease in some cases due to the concerns over the risk to incur in litigation or medico-legal problems. For example, an unplanned splenectomy caused by ISI may be intently explained for reasons of surgical radicality. 3) This is a single-center study, and the patients included in this study are all Chinese. Therefore, the generalizability of the findings to populations with different ethnics, races, or geographical environments, and to surgeons with different levels of surgical experience may be limited. 4) The exact definition of “criminal fold” is not widely recognized, which might, to some extent, weakens the reliability of the conclusions. 5) The most important predictor of ISI is the surgeon’s practice. Since it is difficult to quantitively evaluate surgeon’s practice skill, we did not include relevant variable in the multivariate analysis.

In conclusion, BMI≥25 and total gastrectomy can predict high risk of ISI during radical gastrectomy. Laparoscopic surgery is superior to open gastrectomy in lowing the risk of ISI.

## Data availability statement

The original contributions presented in the study are included in the article/supplementary material. Further inquiries can be directed to the corresponding authors.

## Ethics statement

The studies involving humans were approved by The ethics committee of the second affiliated hospital of naval medical university. The studies were conducted in accordance with the local legislation and institutional requirements. The participants provided their written informed consent to participate in this study. Written informed consent was obtained from the individual(s) for the publication of any potentially identifiable images or data included in this article.

## Author contributions

XZ: Writing – review & editing, Writing – original draft, Funding acquisition, Data curation, Conceptualization. ZW: Writing – review & editing, Validation, Supervision, Methodology, Investigation. HF: Writing – original draft, Supervision, Data curation. ZH: Writing – review & editing, Software, Methodology, Formal analysis. WW: Writing – review & editing, Conceptualization. RY: Writing – review & editing, Conceptualization.
